# BACTIBASE: a new web-accessible database for bacteriocin characterization

**DOI:** 10.1186/1471-2180-7-89

**Published:** 2007-10-17

**Authors:** Riadh Hammami, Abdelmajid Zouhir, Jeannette Ben Hamida, Ismail Fliss

**Affiliations:** 1 Unité de Protéomie Fonctionnelle & Biopréservation Alimentaire, Institut Supérieur des Sciences Biologiques Appliquées de Tunis, Université El Manar, Tunisie; 2 Institut des Nutraceutiques et des Aliments Fonctionnels (INAF), Québec. Université Laval, Canada

## Abstract

**Background:**

Bacteriocins are very diverse group of antimicrobial peptides produced by a wide range of bacteria and known for their inhibitory activity against various human and animal pathogens. Although many bacteriocins are now well characterized, much information is still missing or is unavailable to potential users. The assembly of such information in one central resource such as a database would therefore be of great benefit to the exploitation of these bioactive molecules in the present context of increasing antibiotic resistance and natural bio-preservation need.

**Description:**

In the present paper, we present the development of a new and original database BACTIBASE that contains calculated or predicted physicochemical properties of 123 bacteriocins produced by both Gram-positive and Gram-negative bacteria. The information in this database is very easy to extract and allows rapid prediction of relationships structure/function and target organisms of these peptides and therefore better exploitation of their biological activity in both the medical and food sectors.

**Conclusion:**

The BACTIBASE database is freely available at , web-based platform enabling easy retrieval, via various filters, of sets of bacteriocins that will enable detailed analysis of a number of microbiological and physicochemical data.

## Background

Bacteriocins are antimicrobial peptides produced by many bacteria and display inhibitory activity against closely related bacteria [1]. Bacteriocin production in some bacteria is regulated by a widespread mechanism of the quorum sensing type that appears to play a major role in monitoring the cell density of a bacterial population in a given environment [2].

Since their discovery by [3] in 1925, nearly 300 bacteriocins have been identified and some of them have been used successfully for inhibiting both animal and human pathogens [4,5]. A few of these have been well characterized and information such as amino acid sequence and spectra of antimicrobial activity are now available. However, for many other bacteriocins, this kind of information is still missing or is scattered in the scientific literature and therefore unavailable to potential users. This situation could be improved by a central resource such as a database in which data could be collected, analyzed and used to generate new and useful information.

The majority of sequenced bacteriocins are stored in the manually annotated UniProtKB/Swiss-Prot database and relevant data are available as information about mode of action, 3D-structure data, post-translationally modification of the precursor protein in order to release the mature active peptides, interactions with other proteins, cross-references to external databases and Prosite patterns or profiles. UniProtKB/Swiss-Prot represents a large database with broad domains. Thus, there is a clear need to gather, filter and critically evaluate this mass of information and store into smaller, more specialized, resources so that it can then be used in a way that enhances efficiency. Three different databases have been created for antimicrobial peptides and are mentioned in the literature. Two of these, namely ANTIMIC [6] and APD [7] contain very general information about peptides of all types having antibacterial, antifungal or antiviral activities and originating from either eukaryotic or prokaryotic cells. Bacteriocins are not described with a useful amount of detail in either of these databases. For example, ANTIMIC database contains no information on nisin, one of the most studied and used lactic acid bacterial bacteriocins, while the APD database provides no information about the structure of this bacteriocin. Recently, a new database named BAGEL was created and described [8]. BAGEL represents a genome mining tool that allows detection of putative bacteriocin gene clusters in (new) bacterial genomes. This database contains information based on bacteriocin gene clusters but not on protein properties.

A new database designed specifically for bacteriocins is therefore needed. The microbial physicochemical and structural proprieties provided in such database would allow better and more comprehensive structural and functional analysis of this special group of antimicrobial peptides. This would certainly be useful in food preservation or food safety applications, but also has implications for the development of new drugs for medical use.

## Construction and content

BACTIBASE runs on a Windows NT platform (Microsoft Windows 2000) with the Apache web-server (version 2.0.54), MySQL server (v 5.0.30) and PHP (v 4.3.11). The web server and all parts of the database are hosted at the Centre de Calcul El Khawarizmi (CCK), Tunisia. Bacteriocin peptide sequences were collected from the UniProt database [9] and from the scientific literature using PubMed. Microbiological information was collected from the literature by PubMed search. Since not all known bacteriocin sequences were present in the SRS server of ExPASy [10] and the NCBI server [11], our accumulated knowledge in combination with literature search was used to complete the BACTIBASE sequence database. The Blast program was used for the sequence homology search in the database (BlastP version 2.2.15) [12]. Sequence alignment was done using the ClustalW program (version 1.83) [13]. The Java platform is required for visualizing generated phylogenic trees. The peptides collected in this version of BACTIBASE are mainly from natural sources. Precursor sequences were removed to keep only mature peptide sequences. For each peptide, a unique six-digit identification number (ID) starting with the prefix BAC was assigned. Each entry was checked in the Protein Data Bank (PDB) or UniProt database. A web link in BACTIBASE to UniProt and PDB was created for all peptides that already exist in these databases, to facilitate consultation of the original databases. In addition, each entry contains general data such as peptide name, sequence, class, microbial data (producer organism, taxonomy and target bacterial organisms) and relevant references in Swiss protein database. Additional physicochemical data are provided, including empirical formula, mass, length, isoelectric point, net charge, basic, acidic, hydrophobic and polar residues, hydropathy index, binding potential index, instability index, aliphatic index, half-life in mammalian cells, yeast and *E. coli*, cysteine and glycine content, extinction coefficient, absorbance at 280 nm, absent and most prevalent amino acids, secondary (α-helix or β-strand) and tertiary structure (when available), physical method used for structural determination (e.g. NMR spectroscopy or X-ray diffraction) and critical residues for activity, when information is available.

## Utility and Discussion

### Database description

The database main page contains the following interfaces: About Bacteriocins (introduction), general information (with query interface), physicochemical data (with query interface), user sequence analysis interface, user sequence BLAST interface, statistical data, useful links and Contact information. The query interface provides quick or advanced search with a variety of parameters. Users can find a specific bacteriocin peptide using its ID, name or Swiss-Prot ID, query for lists of bacterial organisms targeted by a bacteriocin or for lists of bacteriocins that target a specific organism. Detailed information for each entry in the database can be viewed by clicking on the bacteriocin name. The advanced search tool allows query of all available data.

When a sequence is entered, the program returns all peptides containing this sequence and search results can be sorted into visible columns. A combinatorial search can be done by query of search results. Files containing the sequence (Fasta format) may be downloaded for all of the entries identified by the query, to facilitate other analyses. Registered users can also download output result tables in XLS and DOC format. Users may also submit their own sequences for physicochemical analysis via an interface with prompts. In addition, BLAST enables users to search the database for homologous sequences and save successful results temporarily in the server for subsequent access. Users may thus select some or all of the homologous sequences for multiple aligning with their submitted sequences. The statistical interface provides data on peptide sequence, function and structure. The average length, net charge and amino acid residue percentages for all entries in the database are also listed, as is the frequency of given values for each physicochemical parameter. For structural analysis, the number of peptides with a defined structural type is shown.

### Statistical description and findings

**Figure 1:**
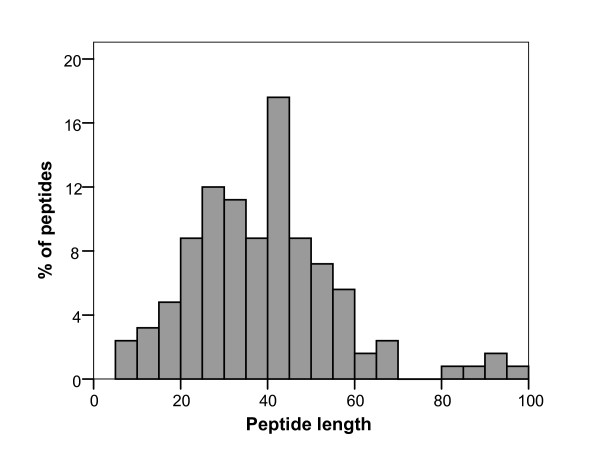
Histogram of the distribution of peptide length in the BACTIBASE database.

**Figure 2:**
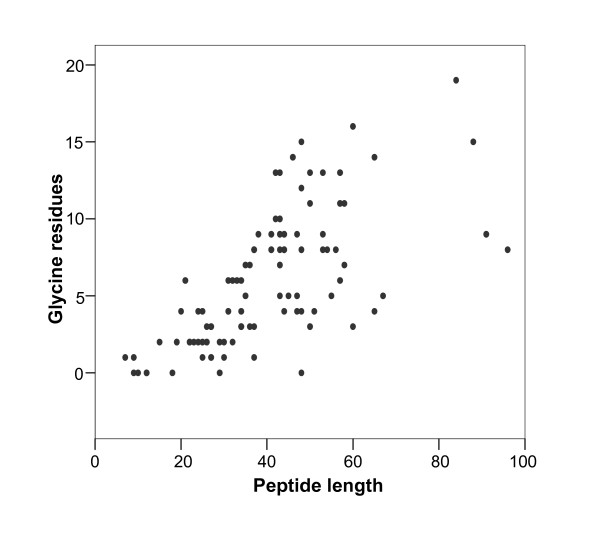
Correlation between length and number of glycine residues among peptides in the BACTIBASE database.

**Figure 3:**
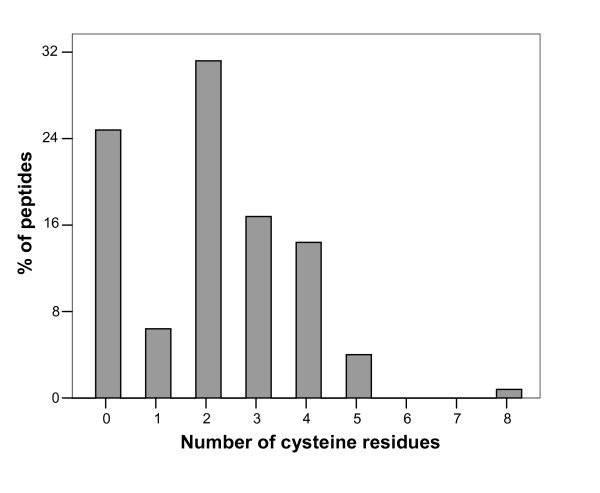
Histogram of the distribution of cysteine residues among peptides in the BACTIBASE database.

**Figure 4:**
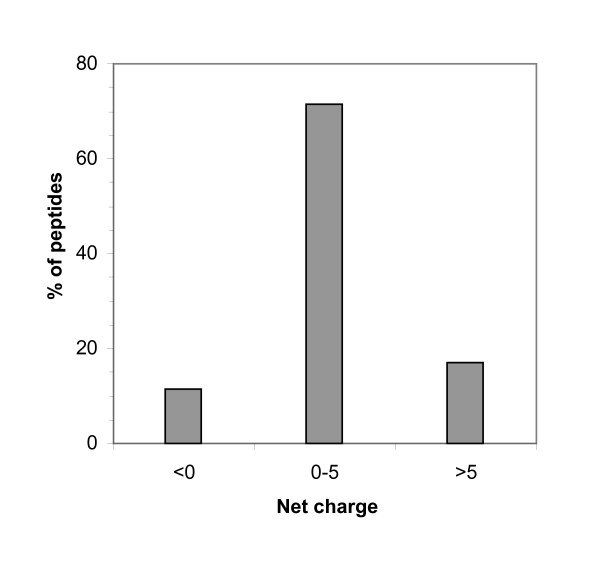
Histogram of the distribution of the net charge among peptides in the BACTIBASE database.

**Figure 5:**
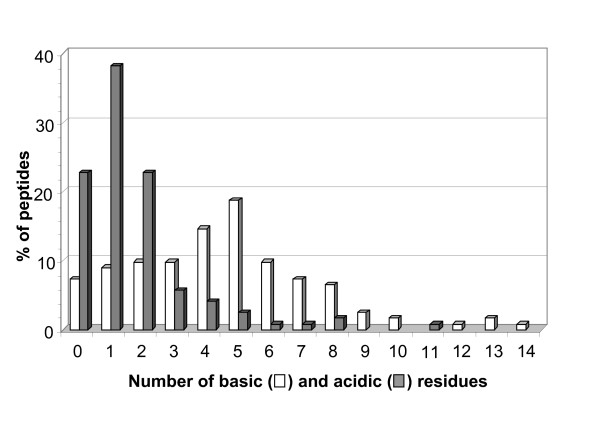
Bar graph of the distribution of acidic and basic amino acids among peptides in the BACTIBASE database.

The current version of BACTIBASE holds 123 bacteriocins, 113 secreted by Gram-positive bacteria and 10 by Gram-negative bacteria. Classification has been proposed on the basis of primary structure [14-16]. Various bacterial species produce bacteriocins. The lactic acid bacteria (order *Lactobacillales*) are the predominant group of producers. Of those in our database, 86 are produced by lactic acid bacteria, two by halobacteria, six by actinobacteria, 18 by bacilli, four by clostridia and seven by proteobacteria. For 81.3%, the amino acid sequence length varies from 20 to 60 (Figure 1). Table 1 summarizes the amino acid percentages. Glycine is the most abundant amino acid and 93.5% of these bacteriocins contain at least glycine residue. Calculated Pearson coefficients (r = 0.635) revealed a positive correlation between sequence length and number of glycine residues, indicating that glycine content is fairly constant (Figure 2). It is noteworthy that 25% of the sequences do not contain cysteine and about 32% contain only one pair, as can be seen in Figure 3. We also note low proline content, with over 74% of the amino acid sequences containing either one residue or none. The majority (71%) of sequences have net charges varying from 0 to +5, less than 18% possess a positive charge superior to +5, with the highest being +12 (BAC107) (Figure 4). In addition, only 11.4% of the sequences have a net negative charge, the most negatively charged bacteriocin having a net charge of -4 (BAC097). As a result, the average net charge of all bacteriocins in BACTIBASE is +2.90. Figure 5 shows the distribution of basic and acidic residues. The majority of sequences display a basic pattern, 43% having from four to six basic residues. In comparison, acidic residue content is more limited. Over 22% do not contain any acidic amino acid and 83.7% contain two or fewer acidic amino acids. Current analysis revealed that three quarters of the bacteriocins contain between five and 20 hydrophobic residues. Hydrophobicity and basicity are major criteria for bacteriocin activity [17]. Only 16 of the bacteriocins were found to have 3D structures filed in the PDB database and resolved by NMR spectroscopy or crystallography. Some of them nevertheless possess many structures in the PDB database, bringing the total number of 3D entries to 24. These findings may be useful in isolating and characterizing novel bacteriocins or designing novel peptides with higher potency against pathogens or with broad antimicrobial spectra.

**Table 1:** Amino acid occurrence in the BACTIBASE database

**Amino acid**	**Number of residues**	**% of total residues**
G (glycine)	717	**15.03**
A (alanine)	491	**10.29**
K (lysine)	360	**7.55**
S (serine)	343	**7.19**
V (valine)	299	**6.27**
N (asparagine)	295	**6.18**
T (threonine)	292	**6.12**
I (isoleucine)	270	**5.66**
C (cysteine)	254	**5.32**
L (leucine)	253	**5.30**
W (tryptophan)	160	**3.35**
Y (tryrosine)	159	**3.33**
F (phenylalanine)	149	**3.12**
P (proline)	130	**2.73**
Q (glutamine)	107	**2.24**
H (histidine)	101	**2.12**
E (glutamic acid)	100	**2.10**
D (aspartic acid)	99	**2.08**
R (arginine)	95	**1.99**
M (methionine)	80	**1.68**
X (variable)	16	**0.34**

As future developments, we plan to integrate a system that will allow automatic prediction of bacteriocin secondary structure, functional amino acids and a server for building tertiary structures by homology with existent bacteriocin structures.

## Conclusion

BACTIBASE allows all bacteriocins sequence and information data to be accessed via a user-friendly, web-based interface. The database can be queried using various criteria, and retrieval, at either microbiological or physicochemical level, includes specific information on each bacteriocin. The provided microbial physicochemical and structural proprieties would allow better and more comprehensive structural and functional analysis of this special group of antimicrobial peptides. BACTIBASE should help enhance our understanding of bacteriocins biology. This would certainly be useful in food preservation or food safety applications, but also has implications for the development of new drugs for medical use.

## Availability and requirements

The BACTIBASE web-server is freely accessible at  for *de novo *searches for bacteriocin-related information. The BACTIBASE database can be queried from this web interface. More options are provided for registered users. The Java platform  is required for visualizing generated phylogenic trees. Researchers in this field are invited to use the database, make suggestions and submit their peptides. All comments, queries, requests and corrections should be sent by email to . Depositions of new sequence entries for naturally occurring bacteriocins should be sent to this address via email and should be accompanied by a reference to a published, peer-reviewed article. Users of BACTIBASE are requested to cite this article when referencing the database. BACTIBASE currently contains 123 entries of bacteriocins and is expected to grow quickly with the rapid development of genomic and proteomic projects. As more information about bacteriocins becomes available, the database will be expanded and improved accordingly.

## Competing interests

The author(s) declares that there are no competing interests.

## Authors' contributions

RH programmed the database and interface, performed the statistical analysis and drafted the manuscript. AZ participated in the design of the study, interacted with RH to carry out the microbiological and physicochemical data and provided specialist knowledge on bacteriocin sequences. RH and AZ would be considered as contributed equally to this work. JBH oversaw the project and helped define user requirements. IF conceived of the study, and participated in its design and coordination and helped to draft the manuscript. All authors read and approved the final manuscript.
